# Risk factors for measles death: Kyegegwa District, western Uganda, February–September, 2015

**DOI:** 10.1186/s12879-017-2558-7

**Published:** 2017-07-03

**Authors:** Richardson Mafigiri, Fred Nsubuga, Alex Riolexus Ario

**Affiliations:** 1 Uganda Public Health Fellowship Program – Field Epidemiology Track, Kampala, Uganda; 2 Uganda Public Health Fellowship Program, Ministry of Health, P.O. Box 7272, Kampala, Uganda

**Keywords:** Measles, Outbreak, Risk factors, Uganda

## Abstract

**Background:**

On 18 August 2015, Kyegegwa District reported eight deaths during a measles outbreak to the Uganda Ministry of Health (MoH). We investigated this death cluster to verify the cause, identify risk factors, and inform public health interventions.

**Methods:**

We defined a probable measles case as onset of fever and generalised rash in a Kyegegwa District resident from 1 February – 15 September 2015, plus ≥1 of the following: coryza, conjunctivitis, and cough. A confirmed measles case was a probable case with measles-specific IgM positivity. A measles death was a death of a probable or confirmed case-person. We conducted an active case-finding to identify measles patients who survived or died. In a case-control study, we compared risk factors between 16 measles patients who died (cases) and 48 who survived (controls), matched by age (±4 years) and village of residence.

**Results:**

We identified 94 probable measles cases, 10 (11%) were confirmed by positive measles-specific IgM. Of the 64 probable measles patients aged <5 years, 16 died (case-fatality rate = 25%). In the case-control study, no history of vaccination against measles was found in 94% (15/16) among the case-persons (i.e., measles patients who died) and 54% (26/48) among the controls (i.e., measles patients who survived) (OR_M-H_ = 12; 95% CI = 1.6–104), while 56% (9/16) of case-persons and 67% (17/48) of controls (OR_M-H_ = 2.3; 95% CI =0.74–7.4) did not receive vitamin A supplementation during illness. 63% (10/16) among the case-persons and 6.3% (3/48) of the controls (OR_M-H_ = 33; 95% CI = 6.8–159) were not treated for measles illness at a health facility (a proxy for more appropriate treatment), while 38% (6/16) of the case-persons and 25% (12/48) of the controls (OR_M-H_ = 2.5; 95% CI = 0.67–9.1) were malnourished.

**Conclusion:**

Lack of vaccination and no treatment in a health facility increased the risk for measles deaths. The one-dose measles vaccination currently in the national vaccination schedule had a protective effect against measles death. We recommended enhancing measles vaccination and adherence to measles treatment guidelines.

## Background

Measles is caused by a virus in the family of paramyxovirus, it is passed on through direct contact and air. Measles virus infects the mucous membranes and spreads all over the body (WHO). It causes enormous number of deaths among the children globally, World Health Organisation estimated about 145,700 people died of measles in 2013 majority being under the age of 5, 95% of the death occurred in politically and health system burdened countries of Africa and Asia [1]. Measles virus is highly contagious and a leading cause of death from vaccine-preventable diseases among children [2, 3]. Uganda like many other developing countries, is still faced with the huge challenge of measles which is a serious public health concern [4]. The influx of refugees from neighbouring countries has compounded the problem further. In 1984, the World Health Organisation (WHO) launched the Expanded Program on Immunization (EPI) in Uganda [5]. The program organizes immunisation of children against the known killer diseases which include; hepatitis B, respiratory diseases due to haemophilus influenza, measles, polio, tetanus, whooping cough, diphtheria, and tuberculosis [5, 6]. Vaccination is the only sure means of guaranteeing safety and survival of children against the vaccine-preventable diseases like measles [5, 6]. Certainly, vaccination remained a crucial arena at attainment of the Millennium Development Goal 4 (MDG4) which was intended to reduce the child mortality by two-thirds by the year 2015 [5]. There is proof that late vaccination is associated with reduced survival as compared to the early one which improves the infant’s life [7].

Despite the efforts and resources invested in the fight against measles outbreaks, there are still a number of them occurring in different parts of the country some resulting into complications [8]. These include encephalitis, diarrhoea, pneumonia, otitis media (ear discharge), these may lead to death of a child if neglected during illness [4]. In Uganda, a single dose of measles vaccine is administered at 9 months mandatory to every child [8]. Vaccine efficacy at that time is expected to be 85%, however, this could be hampered by vaccine failure, cold chain breakage and lack of funds for immunization exercise etc. [4]. However, many children below 9 months are also susceptible to measles infection, the present policy excludes this age. Certainly because it is believed that infants receive antibody protection passed on to them through breast feeding by their mother [9] and in a study by Moss and colleague, it is stated that infants have poor response to measles vaccine [10]. Also in many instances, cultural beliefs inhibit some communities from taking their children for vaccination due to lack of trust on the vaccines used. All this may enhance the risk of measles infection and death from the disease. During this outbreak in Kyegegwa district, the Uganda Ministry of Health was alerted of the high number of deaths which occurred. We conducted this investigation to determine the risk factors for measles death among children below the age of 5 years in this district.

## Methods

### Study area

**Fig. 1:**
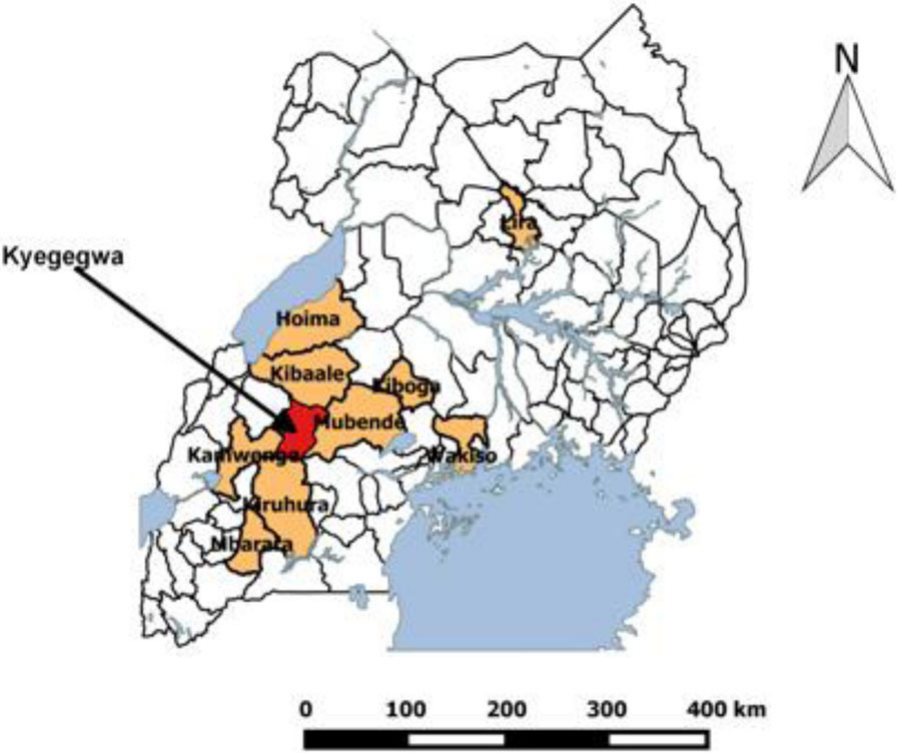
The map of Uganda showing the location of Kyegegwa District in western part of the country

**Fig. 2:**
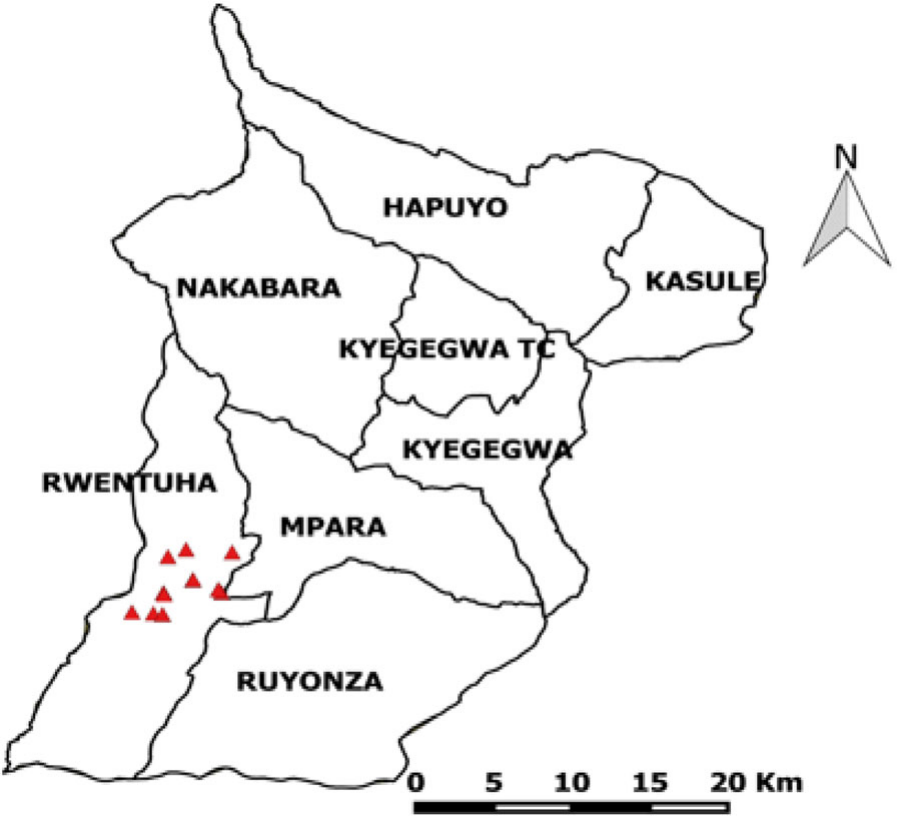
The map of Kyegegwa District showing the location of measles case-persons in Rwentuha Sub-County

Kyegegwa District is located in Western Uganda. It has a total population of 285,328 as of 2014 census consisting of 141,620 males and 143,708 females with an annual growth rate of 11%. The district is bordered by Kibaale in the North, Mubende in the East, Kyenjojo in the North West, Sembabule in south Eastern and Kiruhura in the South. Kyegegwa is approximately 210 km (130 miles) by road from Kampala, the capital of Uganda, Figs. 1 and 2.

The district has a high number of refugee population due to political instability in the neighboring Democratic Republic of Congo (DRC) and Burundi. There are two refugee camps, Kyaka II with approximately 25,000 people and Rwamwanja which hosts approximately 57,000 people. This area (Kyegegwa) is occupied by multi-tribal communities comprising of mainly the indigenous Batooro, Banyankole and Bakiga migrants from other districts. There are also a good number of Banyarwanda, Bakonjo, and others tribes. The main economic activities practiced by the people of the district are; subsistence farming, growing crops on a small scale and keeping small numbers of animals for income generating purposes and food.

### Case definition

We defined a probable measles case as onset of fever and generalised rash in a Kyegegwa District resident from 1 February – 15 September 2015, plus ≥1 of the following: coryza, conjunctivitis, and cough. A confirmed measles case was a probable case with measles-specific IgM positivity; a measles death was death of a probable or confirmed measles case-person.

### Study population

The study population comprised of Kyegegwa District, Rwentuha Sub-County residents who experienced measles outbreak. In the study, the case-persons who met the case definition were purposively selected from the community. Control-persons were selected from the neighbourhood of the cases in the same community.

### Finding and interviewing cases

We worked together with the village heath teams (VHTs) because they are residents in the communities and speak the same language as the residents’ hence making communication easy. We searched for cases from the community and also identified controls. We interviewed mothers of the deceased and surviving children using a standard questionnaire. We collected information on vaccination history of the child, treatment history, visiting health facility during measles illness and demographic characteristics. We obtained verbal consent from the mothers after thoroughly explaining the aim and objectives of the study.

We also reviewed measles surveillance data from DHIS2 to compare with the active surveillance during the outbreak period. DHIS 2 is an electronic Ministry of Health national health information system which is used for - integrated data management and analysis, health program monitoring and evaluation, facility registry functions, etc. Data from health units is captured using HMIS tools and analysis of data is done periodically at national level for reporting and determination of disease trends and alerts.

### Potential risk factors

We identified the potential risk factors from existing literature and proceeded to formulate a questionnaire based on these; vaccination history, treatment history (vitamin A supplementation during measles illness), visiting a health facility and nutritional status. We hypothesised that vaccination status, vitamin A supplementation, inappropriate treatment and nutritional status could have contributed to the high mortality in measles cases.

### Assessment of vitamin a supplementation

We assessed vitamin A supplementation by including questions in our questionnaire interrogating uptake of vitamin A supplement. We asked the caretaker whether the child had ever been given vitamin A at age six month, one year, two years old and others. We further asked whether the child was given vitamin A supplement during measles illness, and if so, how many times vitamin A was given. In Uganda, as in many other sub-Saharan African countries, the programs for vitamin A supplementation are available and mandatory to children between 6 and 59 months of age [11, 12].

### Laboratory investigations

Approximately 5 ml of blood by venepuncture were collected from a suspected measles patient into a sterile tube and labelled with the patient’s identification details and date of collection. The samples were kept in a refrigerator at 4–8 °C before transportation to the Uganda Virus Research Institute for diagnosis. For each sample, a case investigation form was filled and packed together with the corresponding sample in a plastic bag. The well packed samples were then transported for processing as per the guideline [13]. Overall, fourteen blood samples were collected from suspected measles patients in Rwentuha Sub-county. To declare a measles outbreak in Uganda, at least five samples collected in an area in the same period must test IgM positive.

### Case-control study

We conducted a case-control study to test the hypotheses, where we interviewed the parents of 16 case-persons (i.e., mealses probable or confirmed case-patients that died) and 48 control-persons (i.e., measles case-patients that survived) using a structured questionnaire in a ratio of 1:3. We estimated the sample size by assuming a confidence level of 95%, 80% power, 50% exposure of controls to having been vaccinated for measles, given Vitamin A supplimentation during measles illness, being treated at a health facility during measles illness and not being mulnuorished and an odds ratio (OR) of at least 2.5. We excluded all case or control-persons above 5 years of age. This is because, there were no measles deaths that occurred in the age of 5 years and above. Controls were children below 5 who survived measles selected from the neighbourhood in the same village. We asked mothers of case-persons and controls about the vaccination history, treatment history, and whether they obtained treatment from a health facility. We asked for the medical records and vaccination cards for children for guidance about the child’s vaccination history, received proper treatment, and vitamin A supplementation during measles illness We used photos as proxy of normal and malnourished children to get a rough idea whether children who died were malnourished or not. This is because cases-persons were dead children and we could not determine their nutrition status other than use of photos as proxy to guide us. For the controls we assessed the nutritional status during the interview by means of dietary history and assessment of anthropometric parameters where we used weighing scales and MUAC tapes. Also, we assessed malnutrition by use of clinical signs such as oedema for presence of malnutrition. We asked mothers to recall the types of food they normally served their children, amount and frequency in at least 48 h.

### Statistical analysis

We used Microsoft excel and Epi Info7 for analysis. We performed univariate analysis to assess association between risk factors and death in a measles patient. We obtained Adjusted Mantel-Haenszel Odds Ratios (OR_M-H_) and their corresponding 95% confidence intervals (CIs) [14].

### Ethical considerations

The Uganda Ministry of Health gave the directive and approval to investigate this outbreak. The Office of the Associate Director for Science, CDC-Uganda, determined that this activity was not human subjects’ research, and its primary intent was public health practice or a disease control activity (specifically, epidemic or endemic disease control activity). This is in line with the International Guidelines for Ethical Review of Epidemiological Studies by the Council for International Organization of Medical Sciences (1991). Verbal informed consent was obtained from the mothers of the deceased and surviving children involved in the study before the start of the each interview. However, written consent could not be obtained because most of the participating mothers were uneducated. Nonetheless, the purpose of the investigation was explained to the participating mothers. Also participants were informed that their involvement was entirely voluntary and their refusal to respond to any or all of the questions would not result into a penalty of any sort. Participants confidentiality involving personal information were de-identified during data analysis, and the interview forms were locked up.

## Results

### Descriptive epidemiology

Analysis of measles surveillance data from the district health information system 2 (DHIS2) had identified 85 probable measles cases and one confirmed suspected measles death in Kyegegwa district, yet our active case finding identified 94 probable cases and 16 deaths. An indication that more cases in addition to those that were reported in the DHIS2 were identified, especially those who had died.

**Fig. 3:**
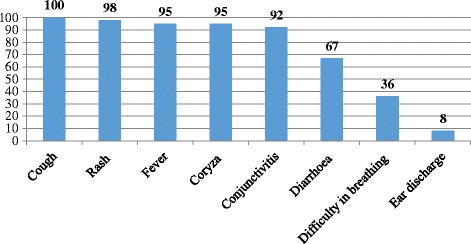
Distribution of measles common symptoms among cases and controls in Kyegegwa District, Feb-Aug 2015

Among all probable measles cases (*n* = 94), 46% (43/94) were females and 54% males. The age range of the probable case-persons was 0.3 months – 36 years; 35% (33/94) were ≤1 year and 68% (64/94) were ≤5 years of age. The case-fatality rate among all 94 probable case-patients was 17% (16/94). All deceased persons were <4 years of age, and the case-fatality rate among those aged <5 years was 25% (16/64). The mean age of the deceased was 1.4 years ranging between 4 months to 3 years. Figure 3 shows symptoms of the deceased persons which included cough (100%), rash (98%), fever (95%), coryza (95%), conjunctivitis (92%), diarrhoea (67%), difficulty in breathing (36%), and ear discharge (Otitis media) (8%).

### Findings of the case-control study

No vaccination against measles was 94% (15/16) among the case-persons and 54% (26/48) among the controls (OR_M-H_ = 12; 95% CI = 1.6–104), while 56% (9/16) of case-persons and 67% (17/48) of controls (OR_M-H_ = 2.3; 95% CI =0.74–7.4) did not receive vitamin A supplementation during measles illness. Among those who did not receive appropriate treatment, 63% (10/16) of the case-persons and 6% (3/48) of the controls (OR_M-H_ = 33; 95% CI = 6.8–159) were not treated for measles illness at a health facility, while 38% (6/16) of the case-persons and 25% (12/48) of the controls (OR_M-H_ = 2.5; 95% CI = 0.67–9.1) were malnourished (Table 1).

**Table 1:** Bivariate analysis for risk factors for measles death for cases and controls in Kyegegwa District, western Uganda, August 2015

	%		
Variable	Cases	Controls	OR_M-H (95% CI)_
	(*n* = 16)	(*n* = 48)	
Never treated at a health facility	63	6	33 (6.8–159)
No measles vaccination	94	54	12 (1.6–104)
No vitamin A supplementation	56	35	2.3 (0.74–7.4)
Malnutrition	38	25	2.5 (0.67–9.1)

*OR*_*M-H*_ Mantel-Haenszel odds ratio, *CI* confidence interval

^a^Fisher’s exact CI

Children who were not vaccinated, who did not receive vitamin A supplementation or were managed from home were significantly more likely to die from measles compared to those who were vaccinated, or received vitamin A supplementation or received treatment from a health facility. Malnutrition was not significantly associated with increased risk of death during measles illness in Kyegegwa.

### Laboratory results

Measles was confirmed in ten of the fourteen samples which were submitted to the Uganda Virus Research Institute for diagnosis.

## Discussion

This investigation has demonstrated that the risk of death from measles during the current outbreak was higher in children who were not vaccinated, managed at home or did not receive vitamin A supplements and malnutrition. Vitamin A supplementation and malnutrition showed high odds ratios indication an association with death, although they were not statistically significant.

Uganda currently administers a one-dose measles-containing vaccine to children at 9 months as part of the national vaccination schedule. This is similar in many sub-Saharan African countries where a routine single dose is administered at 9 month [15]. The administrative coverage for measles in Kyegegwa District is estimated at 79%. This is lower than the recommended vaccination coverage of ≥90% required to achieve population immunity by the WHO African Region [16]. Even in countries where good immunization coverage has been achieved, measles outbreaks still occur because susceptible population still accumulates fairly rapidly even in countries with high immunization coverage of two-dose measles vaccine, as measles vaccine is not 100% effective [17]. However, our investigation demonstrated that the mortality of children with one-dose vaccination was lower than in those not vaccinated, since these children would suffer from milder forms of measles disease. Subsequently, our study found that majority of the deaths were below 9 months. This suggests that there is need to lower the age at which measles vaccination should be given especially in countries like Uganda where measles is an endemic disease.

Malnutrition and vitamin A deficiency are recognised risk factors for severe measles. That is why the World Health Organization (WHO) recommends administration of an oral dose of 200,000 IU (or 100,000 IU in infants) of vitamin A per day for two days to children with measles, especially in areas where vitamin A deficiency may be present [12]. Our investigation showed that malnutrition and lack of vitamin A supplementation had positive (high odds ratios) but not statistically significant associations with measles deaths. This is probably due to an insufficient sample size especially case-persons. Also, difficulty in breathing, diarrhoea and otitis media occurred in a significant proportion of case-persons during this outbreak as shown in (Fig. 3).

Both vitamin A supplementation and management of severe symptoms are better achieved by a trained health worker in a health facility than by lay persons at home. Measles patients managed at home are therefore more likely to die due to complications from measles. This is assumed that at health facilities, clinician adhere to the standard guidelines for measles treatment and management as explained in a study by Pegorie et al. [18]. In a review study conducted by Mayo-Wilson et al. (2011), they concluded that vitamin A supplementation reduces the incidence of death among children <5 years of age with measles [19]. For that matter therefore, there is need to scale up health education to parents in the villages and general population about the benefits for treating measles at a health facility and dangers of measles patients not being managed at health facility for better treatment.

The routine surveillance system in the country report all suspected outbreaks and deaths due to measles to the national level. However, our active case investigation yielded high numbers of deaths compared to those that had been reported in the national surveillance system. In line with our study, Anand et al. (2009) in a study measles deaths in Nepal also found that measles deaths and cases were being under reported by the routine surveillance system [20]. This could be probably due to the fact that most deaths occurred at home, and hence were not captured by the system as there is lack of systematic procedures to report such incidences at local level or villages even in the presence of the village health teams. This may also be blamed onto the local people with cultural perception that any measles patients should be treated locally with local herbs not in the hospital. There is need to strengthen the village health teams’ capacity for surveillance and reporting.

### Limitations of the study

Due to a relatively small number of deaths, the sample size in our investigation was only able to detect strong associations. Lack of sufficient sample size also rendered it impossible to conduct more detailed analysis (i.e., stratified analysis and regression analysis) to control for confounding. The risk factor data were mainly based on self-reporting, leaving the estimated associations vulnerable to potential information bias. Also, non-availability of vaccination records, hence almost of all information obtained was verbal. Recall bias is a possibility here, there were hardly any medical records to authenticate vitamin A supplementation during measles illness. Also use of pictures to assess the nutritional status of the deceased children may not have generated accurate information as some mothers would not be comfortable admitting that their children died because they were malnourished.

## Conclusion

In conclusion this investigation found that the risk of death was exacerbated by inappropriate treatment (not being treated at health facility), and low vaccination status, during measles illness. The national surveillance system did not pick all the cases and death which active surveillance found. Younger age was a risk factor for mortality. We recommended enhancement of measles vaccination amongst under five year old children. We also recommended management of all measles patients in a health facility for better treatment outcomes. We further recommended two doses of measles vaccine at 6 months and 9 months of age; and strengthening the community component of the Health Management Information System for more complete and accurate reporting.

## Abbreviations

CDC: Centers for Diseases Control and Prevention
DHIS2: District Health Information Software version 2
MoH: Ministry of Health
UVRI: Uganda Virus Research Institute

## References

[1] WHO warns that progress towards eliminating measles has stalled [http://www.who.int/mediacentre/news/releases/2014/eliminating-measles/en/]. Accessed 5 April 2017.

[2] Izadi S, Zahraie S-M, Sartipi M (2012). An investigation into a measles outbreak in southeast Iran. Jpn J Infect Dis.

[3] Mahamud A, Burton A, Hassan M, Ahmed JA, Wagacha JB, Spiegel P, Haskew C, Eidex RB, Shetty S, Cookson S: Risk factors for measles mortality among hospitalized Somali refugees displaced by famine, Kenya, 2011. *Clin Infect Dis* 2013:cit442. 10.1093/cid/cit442 23821730

[4] Akramuzzaman SM, Cutts FT, Hossain MJ, Wahedi OK, Nahar N, Islam D (2002). Measles vaccine effectiveness and risk factors for measles in Dhaka, Bangladesh. Bull World Health Organ.

[5] Bbaale E (2013). Factors influencing childhood immunization in Uganda.

[6] Abdulraheem I, Onajole A, Jimoh A, Oladipo A (2011). Reasons for incomplete vaccination and factors for missed opportunities among rural Nigerian children. JPHE.

[7] Fadnes LT, Nankabirwa V, Sommerfelt H, Tylleskär T, Tumwine JK, Engebretsen IM, Group P-ES: Is vaccination coverage a good indicator of age-appropriate vaccination? A prospective study from Uganda. Vaccine 2011, 29(19):3564–3570. 10.1016/j.vaccine.2011.02.093 21402043

[8] Anis-ur-Rehman ST, Idris M. Clinical outcome in measles patients hospitalized with complications. J Ayub Med Coll Abbottabad. 2008:20(2). 19385448

[9] Maria M, Elena C, Vassiliki P (2011). **Reduced measles and varicella passive immunity and susceptible infants in the 21ST century. Myth or reality?**. AJBS.

[10] Moss WJ, Scott S (2009). WHO immunological basis for immunization series module xx: measles.

[11] Wirth JP, Petry N, Tanumihardjo SA, Rogers LM, McLean E, Greig A (2017). Vitamin a supplementation programs and country-level evidence of vitamin a deficiency. Nutrients.

[12] Guideline: vitamin A supplementation in infants and children 6–59 months of age [http://apps.who.int/iris/bitstream/10665/44664/1/9789241501767_eng.pdf]. Accessed 8 April 2017.

[13] Manual for the laboratory diagnosis of measles viral infection [http://www.measlesrubellainitiative.org/wp-content/uploads/2013/06/Manual-Laboratory-Diagnosis-Measles-Virus-Infection.pdf]. Accessed 4 April 2017.

[14] Fleiss JL, Levin B, Paik MC (2013). **Statistical methods for rates and proportions**: John Wiley & Sons.

[15] Grais R, Dubray C, Gerstl S, Guthmann J, Djibo A, Nargaye K (2007). Unacceptably high mortality related to measles epidemics in Niger, Nigeria, and Chad. PLoS Med.

[16] African Regional Guidelines for Measles and Rubella Surveillance [www.afro.who.int/index]. Accessed 27 December 2016.

[17] Marin M, Nguyen H, Langidrik J, Edwards R, Briand K, Papania M (2006). Measles transmission and vaccine effectiveness during a large outbreak on a densely populated island: implications for vaccination policy. Clin infect dis: an official publication of the Infectious Diseases Society of America.

[18] Pegorie MSK, Welfare WS, Wilson RW, Khiroya C, Munslow G, Fiefield D (2014). Measles outbreak in greater Manchester, England, October 2012 to September 2013: epidemiology and control. Euro Surveill.

[19] Mayo-Wilson E, Imdad A, Herzer K, Yakoob MY, Bhutta ZA (2011). Vitamin a supplements for preventing mortality, illness, and blindness in children aged under 5: systematic review and meta-analysis. BMJ.

[20] Anand B, Joshi ETL (2009). Robin Nandy, Bal K Subedi, Jayantha BL Liyanage & Thomas F Wierzba **Measles deaths in Nepal: estimating the national case–fatality ratio**. Bull World Health Organ.

